# Recurrent thyrotoxic periodic paralysis with normokalemia in a 36‐year‐old man: A case report

**DOI:** 10.1002/ccr3.4849

**Published:** 2021-09-22

**Authors:** Sara Seife Hassen, Mohamed Salih Ali, Adeel Khan, Areej Marwan Mohammed, Mohamed Altermanini, Dabia Hamad S. H. Al Mohanadi, Mohamed Zahid

**Affiliations:** ^1^ Department of Internal Medicine Hamad General Hospital Hamad Medical Corporation Doha Qatar

**Keywords:** acute medicine, endocrinology and metabolic disorders, hyperthyroidism, muscle weakness, paralysis

## Abstract

Among patients with thyrotoxicosis and proximal muscle weakness, some patients with TPP may present with apparent normokalemia in whom a careful administration of potassium may lead to rapid reversal of muscle weakness.

## Graphical abstract

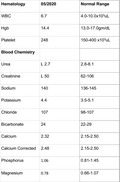

## 1 INTRODUCTION

Thyrotoxic periodic paralysis (TPP) is a rare complication of thyrotoxicosis, presenting with reversible acute paralysis and usually subnormal serum potassium levels. We present a 36‐year‐old man with uncontrolled Graves’ disease who presented three times to our emergency department over a year with recurrent profound acute weakness and normokalemia.

Thyrotoxic periodic paralysis (TPP) is a rare but serious complication of thyrotoxicosis most common in Asian populations, with an incidence of approximately 2 percent in patients with thyrotoxicosis of any cause.^1^, ^2^ 95 percent of TPP cases occur in men.^3^ Although the majority of cases of thyrotoxicosis associated with TPP are due to Graves’ disease, TPP can appear with thyrotoxicosis of any origin. Cases of TPP have been reported with thyroiditis, toxic adenoma, or toxic nodular goiter.^4^ TPP is commonly misdiagnosed in western countries perhaps because of its rarity in these regions of the world, subtle signs of hyperthyroidism on presentation, and its similarities to familial periodic paralysis (FPP). The prevalence of TPP in non‐Asian populations is 0.1–0.2 percent, or about one‐tenth of the prevalence seen in Asian countries.^5^ Thyrotoxic period paralysis classically presents with episodes of acute muscle weakness and paralysis, usually provoked by exercise and change in diet. Rare but fatal complications such as respiratory failure and arrhythmias may occur.^6^ Early diagnosis and management of hypokalemia and hyperthyroidism lead to favorable outcomes.^4^ We present a case of a young male with Graves’ disease who developed TPP in the presence of normokalemia.

## 2 CASE SUMMARY

A 36‐year‐old man presented to the emergency department with weakness in both legs for three hours. He experienced the weakness upon awakening from sleep, of sudden onset and most pronounced in the proximal muscles. The patient also complained of palpitations, tremors in his hands, sweating, and heat intolerance. He denied weakness in any other part of the body, back pain, trauma to the back, sensory loss, urinary retention, constipation, slurring of speech, headache, dizziness, altered level of consciousness, jerky movements, and change in vision or hearing. He had a large carbohydrate meal the night before admission. There was no history of upper respiratory tract infection or diarrhea in the few weeks before presentation. Past medical history was significant for Graves’ disease for one year and an episode of thyrotoxic periodic paralysis secondary to Grave's disease with hypokalemia. He had no family history of thyroid disease or periodic paralysis. He was prescribed carbimazole 20 mg twice daily and propranolol 10 mg twice daily to which he was poorly compliant. The patient had a drug reaction of pruritic rash to carbimazole, which was managed with antihistamines.

On examination, the patient's pulse rate was 78/min and blood pressure 120/75; he was afebrile and maintaining 98% oxygen saturation on room air. He appeared anxious and had bilateral hand tremors, more pronounced on outstretched arms. There was no thyromegaly or tenderness on neck palpation. His pupils were equal and reactive to light, and there was no lid lag, lid retraction, or exophthalmos bilaterally. There was no external ophthalmoplegia. Neurologic examination showed bilateral lower limb weakness with hip flexion/extension 3/5, ankle dorsiflexion 5/5, and plantarflexion 4/5. Knee jerk, ankle jerk, and plantar reflexes were normal bilaterally. Cranial nerve and sensory examination were normal. The cardiovascular, respiratory, gastrointestinal, and genitourinary examinations were unremarkable.

Laboratory investigations showed a normal complete blood count, kidney function tests, liver function tests, urea, creatinine, and serum electrolytes (Table 1). Thyroid function tests showed low TSH, high t3 and T4 levels (Table 2). ECG showed sinus rhythm. The patient was diagnosed with thyrotoxic periodic paralysis, and intravenous potassium 40 mmol in 1 L of normal saline was administered. He had a complete resolution of lower limb weakness within 12 h. The patient was discharged on propylthiouracil 100 mg twice daily and propranolol 80 mg once daily with follow‐up in the endocrine clinic. He was given education about compliance to medication as his compliance was doubtful, evidenced by an uncontrolled thyroid panel, and follow‐up for radioiodine ablation was arranged.

**TABLE 1:** Hematological and electrolyte panel of the patient

Hematology	05/2020	Normal Range
WBC	6.7	4.0–10.0x10^3^uL
Hgb	14.4	13.0–17.0 gm/dl
Platelet	248	150–400 × 10^3^ul
Blood Chemistry		
Urea	L 2.7	2.8–8.1
Creatinine	L 50	62–106
Sodium	140	136–145
Potassium	4.4	3.5–5.1
Chloride	107	98–107
Bicarbonate	24	22–29
Calcium	2.32	2.15–2.50
Calcium Corrected	2.48	2.15–2.50
Phosphorus	1.06	0.81–1.45
Magnesium	0.78	0.66–1.07

**TABLE 2:** Thyroid function test in three presentations of the patient with lower limb weakness

Thyroid Function	05/2020	03/2020	05/2019	Normal Range
TSH	0.01 mIU/L	<0.01 mIU/L	0.01 mIU/L	0.30–4.20
FT3	13.4 pmol/L	30.3 pmol/L	30.6 pmol/L	3.7–6.4
FT4	37.2 pmol/L	54.1 pmol/L	60.3 pmol/L	11.6–21.9
Anti‐thyroid peroxidase antibody			69 IU/ml	0–34
Antithyroglobulin antibody			874 IU/ml	0–115
Anti‐TSH receptor antibody	3.8 IU/L			Cut‐off 1.75 IU/L Positive > = 1.75 IU/L

Within the year before the current admission, the patient had two presentations with sudden onset profound bilateral lower limb weakness (Tables 1, 2). The first presentation was one year before the current admission when he presented with bilateral lower limb weakness upon awakening from sleep, power was 1/5 in the lower limbs and 4/5 in the upper limbs, and there was a mildly diffuse thyroid gland on palpation. His heart rate was 87 and sinus, blood pressure 107/62, no exophthalmos was noted, but he had fine hand tremors bilaterally. His potassium was found to be 1.7, TSH 0.01, and free T4 was 60. Non‐enhanced CT scan of the head was done and ruled out intracranial abnormalities. He was diagnosed with hypokalemic thyrotoxic periodic paralysis and received intravenous potassium replacement. He had total resolution of his weakness within 12 h. Investigations revealed anti‐thyroid peroxidase antibody and antithyroglobulin antibody were positive; a radioiodine uptake scan was arranged. He was discharged on carbimazole 20 mg twice a day and propranolol 10 mg twice a day. He underwent a thyroid scan which showed moderate symmetrical enlargement of the thyroid gland with diffuse uptake of radiotracer, supportive of Grave's disease.

The patient was not compliant with his medication. He stopped taking carbimazole due to a generalized pruritic rash without consulting medical advice. He had a second attack of lower limb weakness shortly thereafter, six months after his initial diagnosis. He was found to have bilateral handgrip 4/5 and lower limb weakness 3/5 with a potassium level of 3.7, TSH 0.01, and free T4 was 57 on laboratory testing (Table 1). He was given Intravenous potassium 40 mmol in 1 L of normal saline over 6 h under strict cardiac monitoring and potassium check for every 4 h. His weakness resolved with potassium administration. Carbimazole was restarted and radioiodine ablation was planned in one month's time. The rash improved with antihistamines and reduced dose of carbimazole to 20 mg oral daily. He was considered for radioiodine ablation one month before his latest admission to our care. The patient still had evidence of thyrotoxicosis upon this third presentation, likely due to non‐compliance to medication.

## 3 DISCUSSION

Thyrotoxic periodic paralysis is a rare complication of thyrotoxicosis, occurring more commonly in Asian men.^7^ Incidence of TPP was 1.8% and 1.9% in China and Japan respectively compared with 0.1% in North America.^8^ The mechanism of hyperthyroidism‐induced periodic paralysis is still unclear, yet the Na/K/ATPase channel has been implicated in the mechanism. Thyroid hormone excess leads to overactivity of the Na/K/ATPase channel, resulting in shifts of potassium toward the intercellular space inducing hypokalemia. Similarly, high insulin states such as following a high carbohydrate diet also activate the Na/K/ATPase activity, leading to hypokalemia. Hyperpolarization of the cell membrane then leads to paralysis due to an in‐excitability of the muscle fibers and precipitating an acute attack of TPP. Other precipitating factors include infection, cold exposure, trauma, menses, emotional stress, pulse steroid therapy, and alcohol ingestion. Diurnal variation and seasonal variability have also been observed, with attacks being more frequent in evening and night, and during the summer season, but the underlying mechanism remains unknown.^9^, ^10^ Our patient was an Asian male, and the episode of paralysis was precipitated by a large carbohydrate meal.

Three other cases have been reported in recent years of TPP in association with normokalemia. The first describes a 26‐year‐old Black man presenting with bilateral lower limb weakness.^11^ He was found to have an undetectable TSH level with an elevation of free thyroxine. One liter of normal saline was administered, with a resolution of the patient's weakness. The second case describes a 33‐year‐old Taiwanese man with upper and lower bilateral limb weakness and suppressed TSH and elevated free T4; he had a potassium level within normal limits. The patient was administered intravenous potassium with improvement in his limb weakness.^12^ The third case is a 19‐year‐old woman with unilateral foot drop and normal potassium level, who was managed with thyroid suppressive medication alone, with a resolution of her neurologic symptoms after control of the thyroid hormone excess.^13^ The timeline of these patients’ recovery from weakness varied from hours to weeks. In contrast to the above cases, our patient had recurrent limb weakness associated with thyroid hormone excess. However, current presentation was associated with normal apparent potassium levels (Table 1). Our patient has normal apparent potassium levels on two different presentations calling into question the diagnosis and requirement of hypokalemia in making the diagnosis of TPP. Classical cases with hypokalemia and TPP have reported successful resolution of muscle weakness after treatment with oral potassium,^14^ although in our case and the other case reports, we have included in our manuscript on normokalemia and muscle weakness note intravenous potassium administration.

TPP is characterized by recurrent transient attacks of muscle weakness, more commonly in the proximal than distal muscles. Leg weakness is more common than arms and it varies from mild weakness to flaccid paralysis.^14^ Our patient on all three occasions had more proximal weakness than distal and more leg weakness than arms.

Familial periodic paralysis (FPP), which also presents with episodic muscle weakness, is an autosomal dominant disorder caused by a defect in the gene coding for L‐type calcium channel 1‐subunit (CACNA1S) on chromosome 1q31–32.^15^ With a prevalence of 1:100,000, FPP has a predilection for males than females, and manifests in the second decade of life.^7^ TPP must be differentiated from FPP which is more common in Caucasian population and patients with a family of periodic paralysis.^5^ Our patient was an Asian male and had no family history of periodic paralysis, and had clinical and biochemical features of hyperthyroidism suggesting the diagnosis of TPP rather than FPP.

The profound paralysis did not correlate with the severity of clinical features of hyperthyroidism. Wayne's Index aids in assessing overt signs and symptoms of thyrotoxicosis with scores less than 11 indicating euthyroid, and 19 and above implying frank thyrotoxic state.^16^, ^17^ Our patient had a score of 17. Our patient's Wayne score was equivocal, supporting further that patients with TPP might not show overt signs and symptoms of excess thyroid hormone.

Without achieving a euthyroid state, the recurrence rate reaches 62 percent within the first 3 months of diagnosis. Once a euthyroid state is reached, periodic paralysis attacks usually do not recur.^18^ Our patient never had biochemical evidence of achieving a euthyroid state (Table 2), which is likely the risk factor for recurrent muscle weakness.

## 4 CONCLUSION

In patients with thyrotoxicosis presenting with proximal muscle weakness, a diagnosis of TPP should be considered after ruling out other etiologies. Although TPP is usually associated with hypokalemia, patients can present with apparent normokalemia as well. A careful administration of potassium even in apparent normokalemic patients can lead to rapid reversal of muscle weakness due to presence of potassium abnormalities at muscle cellular level. Definitive treatment, however, is to achieve a euthyroid state.

## CONFLICTS OF INTEREST

The authors have no conflicts of interest to disclose.

## AUTHOR CONTRIBUTIONS

SH and MA had done history and physical, literature review, and manuscript writing; SH obtained the informed consent; MSA, AK, and AM had done data collection, literature review, and manuscript writing; DM determined eligibility, manuscript review, and manuscript approval; MZ determine eligibility, manuscript writing, and literature review.

## ETHICS APPROVAL

This case report was approved by the Medical Research Center at Hamad Medical Corporation.

## CONSENT

Written informed consent was taken from the patient before the submission of the manuscript.

## Data Availability

Data sharing is not applicable to this article as no new data were created or analyzed in this study.
